# Fulton County Medical Society, Regular Meeting June 3, 1915

**Published:** 1915-06

**Authors:** R. R. Daly


### FULTON COUNTY MEDICAL SOCIETY.

Regualr Meeting June 3, 1915.

R. R. DALY, M. D.

Dr. L. M. Gaines presented a case of Filaria Sanguinous Hominis.

A Barnadoes negro about 30 years old, gave a history of the onset of the disease about 12 years ago. Since that time he has had periodic swelling in the groin and pain in the leg. He would be in bed about one day in the month because of it. He came here for the climate but did not improve during the last two years. He is now in poor general health and presents a swelling that is getting somewhat smaller than it was a few days ago in the right groin.

The diagnosis was made and confirmed by the finding of the parasites in the blood. The condition is rare in this country and is shown on that account. .

Dr. A. H. Bunce said that he had examined the man and had taken samples of his blood at various times but it was not until aften ten o'clock at night that he could get a sample containing the parasite. In a large drop of blood they are plainly to be seen with 2-3 objective and they are about 270 microns long and 17 to 20 wide. They squirm all over the plate until it gets a bit dry and then they seem to be fastened at one end and thrashing about with the rest of the body. They are conveyed by either the anopheles or culex mosquitos. The insects become infected in their own muscles and eventually the parasites reach the probosces and are injected in to the human host. There they are taken up by the lymphatic system and developed until large enough to be conveyed to the blood by means of the thoracic duct. They can be seen in numbers from 2,000 to 5,000 in a c.c. of blood when taken at night. The periodicity may be a week or a month and the host may have been infected 18 years before they show.

Dr. J. L. Campbell said that this disease is common among the negroes of Charleston, S. C. It is called the Demarara groin because of the frequency of the disease in that country.

The swelling usually come in the lymphatics of the groin and the diagnosis is obscure unless this infection is kept in mind. If the patients work at night and rest during the day, the parasites may be found at about ten in the morning in his experience. Removal of the tumors does not accomplish much unless the parent worm be found and there may be more than one of these. They are hermaphrodites and about 4 inches olng and as large as a hair. Chylurea and blocking of the spermatic cord have been caused by the disease. He noted that methyl blue killed them in the laboratory and he thinks it possible that this might be of avail in treating the patient. There is no established treatment.

Dr. M. T. Davis presented a case of Sensitized Vaccines in the treatment of Chronic Eczema. .

We are all familiar with the part played by the Staphylococcus in such surface infections as Furunculosis and Acne. During the past six months my attention has been arrested from time to time, by articles appearing in the Journals, bearing upon the relation between the Staphylococcus and Chronic Eczema.

The patient before you came to me thirteen days ago, with both hands and arms, half way to the elbows, a mass of crusty sores, constantly exuding a sero-purulent secretion, with itching, burning and pain,—the moist type of Eczema, the condition having existed unabated for twenty-two months. A stained smear from the lesions showed great number of Staphylococci, and as the cost of an autogenous vaccine was a consideration, I determined to use Sensitized Vaccines. These, as you remember, are suspensions of killed bacteria, which have been sensitized by treatment with the serum from an animal immunized against the same kind of infection, during which process the bacteria unite with the antibodies contained in the serum, and become saturated with them.

When these killed bacteria, saturated with antibodies, are injected into a patient, they carry these antibodies to the tissue cells, thus sparing them the manufacture of antibodies, which, as we know, are the agents that not only destroy the bacteria, but prepare for phagocytosis the living organisms cauisng the infection.

This patient received four doses of a polyvalent sensitized staphylococcus vaccine, at forty-eight hour intervals, with the gratifying results which you see tonight, his hands and arms being practically free from the trouble for which he consulted me.

The program for the evening comprised the following papers:

I "Twilight Sleep," Dr. W. F. Shallenberger.
II "Uterine Stimulants," Dr. J. R. McCord.
III "Pelvimetry," Dr. O. H. Matthews.
IV "Indications for Forceps," Dr. S. T. Barnett.
V "Caesarian Section," Dr. L. Sage Hardin.

The papers on Twilight Sleep, by Shallenberger, and on Uterine Stimulants by McCord, appear elsewhere in the Journal.

Dr. O. H. Matthews gave a practical review of the diameters of the pelvis calling attention to the importance of systematic pelvimetry and giving the latest and best methods of taking the external and internal measurements. He made special reference to the proper interpretation and significance of minor deviations from the normal.

Dr. S. T. Barnett, in speaking of the forceps, said that they are abused in two ways. A man who is not thoroughly familiar with the pelvimetry of his patient or who waits too long, keeping woamn in labor for many hours or a few days is not doing right in declining the forceps. On the other hand a man who is in a hurry for other reasons or who wants to make a display will use the forceps before the mother has had her chance and he is likely to do unnecessary damage. In all cases a complete and accurate knowledge of the mechanics of labor must be part of the operator's equipment if he is to succeed. High forceps are seldom indicated and have been used altogether too much in the past. Two general heads cover the subject: First, the mother is to be helped when she is dangerously exhausted and the low forceps are to be used to relieve her. Second, the foetus may be asphyxiated or otherwise in danger and it should have the immediate benefit of mechanical assistance. Uterine inertia is a problem at any time as are also flattened and deformed pelves. The forceps may be applied if the os is dilated but not otherwise; then section is to be resorted to. Forceps must never be used to dilate the cervix. Tears and even rupture of the uterus have resulted from this error. Haemorrhage before labor demands instant delivery. If the head is engaged, use forceps; if not engaged, use section.

Dr. L. Sage Hardin, speaking of Caesarian Section, said that high forceps are dangerous for all the reasons Barnett advanced and because one can not control the infection that is pretty sure to follow the injuries to the parts. When there are deformities or repeated haemorrhages or decapitations of children, Caesarian Section should be prepared for in advance and incidentally. it would be well to tie the tubes when the opportunity arose. Section reduces mortality when well done in eclampsia as well as in labor. It is far better to do this than to let the woman die trying to work her way out unaided. The operation itself is no more difficult than hysterectomy and should be approached with no greater alarm. It should be done steadily and with all reasonable speed, but there is no hurry. The uterus should be delivered through the wound and then the child taken from it. If pains have begun it is better because one has not only the tonic of contractions, but there is likely to be some dilatation of the cervix for drainage If this drainage is not present. hysterectomy is indicated and imperative. The technic of the operation is too well known to have elaborate discussion. The after treatment is that of any laparotmy and no ergot may be given.

Dr. J. G. Earnest in discussing the papers said that he had done many high forceps cases and never had failed in them. He never had wounds and though always ready for Caesarian Section, he never had had occasion to use it. He has never done one in his life which has been filled with other surgical work. He chloroforms his patients, sterilizes his hands and goes to work. The hand measures the pelvis and it assists in dialatation when necessary. It goes into the uterus and locates the head beyond the peradventure of a doubt; and oftimes this adjustment hastens and eases the labor. In placenta previa and deformity, section is indicated but he has never been driven to it.

Dr. W. A. Crowe said he had been working in obstetrics all his life and the diagnosis of the position of the child was still hard to make by the ordinary landmarks. He used the lobe of the ear, himself and this has been more satisfactory than the openings. High forceps are dangerous in his opinion and are to be used only after a few indications. Turning and delivering is better whenever the os is dilated and it can be done with splendid results. It is his impression that the process is not used as much as formerly, but it is an excellent one. In Eclampsia one may do either a vaginal or abdomenal section. There is little danger in non-infected cases if one has good assistance. Haemorrhage is controlled by pressure on the ovarian arteries as Hardin has said. Twilight sleep has taken us off our feet. It has not been satisfactory in his few cases and does not appeal to him; it is too complicated.

Dr. T. B. Armstrong said that he was not favorably impressed with the 30 cases he had had at Grady Hospital during the last two years. In 5 there was successful amnesia while in the others there was considerable delirium and excitement. The conditions were not the best, however.

Dr. J. L. Campbell said he had 16 cases in private practice. In 6 there was complete amnesia, in another 6 it was partial and the remaining group were not favorably affected. No really bad results followed. Two cases were excited and had to be restrained which spoiled his asepsis. The children were rather quiet but all did well. He began with the four-minute pains and never gave more than three doses. His long case was 5 hours; short one, one and one-half hours.

Dr. John F. Denton said that in the Grady Hospital cases there were other things contributing to lack of success. Some of these were low forceps cases and many were restless for psychic reasons. One case of persistent posterior caused only a slight tear.

The patients, especially the primiparae seemed to like it and they are saved from the terrors the neighbors have inspired by their talk. Twilight sleep should not be used in two-hour cases in his judgment. Shallenberger's cases show that it should not be condemned, Pituitrin is of value and often does away with low forceps cases. The use of high forceps upon the floating head is obsolete.

Dr. R. R. Kime said that while he had given up obstetrics he had observed the later ideas and he felt they were fads and fashions in the work. They will doubtless find their places soon. In county practise, neither Twilight nor Section would do. Section is a capital operation and must be so approached. Personally, he prefers high forceps and a live mother to death after manipulation and section. As to Stimulants Pituitrin has its place and should be given just as one would administer ergot.

Dr. E. L. Griffin asked what one was to do with a wedged head in delayed labor. Suppose you are out in the country where you can get neither assistance or craniotomy instruments, what will you do? One can not leave the woman undelivered. He puts on forceps and delivers somehow. In such a case you can't put in your hand to turn or to feel ears or anything else that is recognizable for certain. Save the woman even if the child has to die and go ahead with your knowledge and your common sense.

Dr. Hansell Crenshaw remarked that since hyoscin and scopolamin have decided mental effects it might be well to inquire as to possible psychoses in the mother's history before administering these drugs. They have been abandoned in treating habitues of other drugs because of the hallucinations they produce themselves.

Dr. Archibald Smith said that he has long used these drugs though possibly not with the same technic as at Freiburg. They have been of value in operating upon vesico vaginal fistula.

Dr. C. M. Curtis said that there could still be hope in the minds of younger men that they could get along in their obstetrics without having to be frightened about these new and dangerous things. He had in his long practise confined about 1,400 women, mostly in country districts and he long since discarded drugs and allowed nature to take her course with such help as the accoucheur himself could give. He has never used high forceps and has never needed a Caesarian section. It is true that he has lived where he could easily get assistance if he needed it, but after all, knowledge of the conditions and experience in handling them gave good results.

Dr. T. C. Davison said that we should come to some decision as to the use of high forceps in eclampsia and also as to Twilight sleep. He thinks that the Daemmerschlaff should always be staged in a hospital and we must remember that there is not actual absence of pain but loss of recollection of it. He has had 14 cases in 8 months and is undecided abotu it. It requires constant attention and frequent tests of the memory.

